# Ceruloplasmin activity and iron chelation treatment of patients with Parkinson’s disease

**DOI:** 10.1186/s12883-015-0331-3

**Published:** 2015-05-06

**Authors:** Guillaume Grolez, Caroline Moreau, Bernard Sablonnière, Guillaume Garçon, Jean-Christophe Devedjian, Sayah Meguig, Patrick Gelé, Christine Delmaire, Regis Bordet, Luc Defebvre, Ioav Z Cabantchik, David Devos

**Affiliations:** Department of Medical Pharmacology, Lille Nord de France University, Lille, France; Department of Movement Disorders and Neurology, Lille Nord de France University, Lille, France; INSERM U1171, Lille Faculty of Medicine, Lille Nord de France University, Lille, France; Department of Molecular Biology and Pathology Centre, Unit 837, Team 1, INSERM Lille Faculty of Medicine, Lille University Hospital, Lille Nord de France University, Lille, France; Department of Toxicology, Public Health and Environment, EA 4483, Faculty of Pharmaceutic and Biological Sciences, Lille Nord de France University, Lille, France; Biological Resources Centre, Lille University Hospital, Lille, France; Department of Neuroradiology, Lille University Hospital, Lille, France; Della Pergola Chair, Alexander Silberman Institute of Life Sciences, Hebrew University, Jerusalem, Israel

**Keywords:** Parkinson’s disease, Iron chelation, L-dopa, Motor response, Deferiprone

## Abstract

**Background:**

Growing body of evidence suggests that Parkinson’s disease (PD) is associated with oxidative damage via iron accumulation in the substantia nigra (SN). Low ceruloplasmin (CP)-ferroxidase activity has been identified in the SN and the cerebrospinal fluid (CSF) of patients with PD. The iron chelator, deferiprone, reduces the abnormally high levels of iron in the SN. In order to determine CP’s involvement in iron accumulation in SN and PD progression, we aim to compare the ability of iron chelation treatment to reducing both SN iron levels and motor handicap in PD patients according to the level of ceruloplasmin activity.

**Methods:**

We used a moderate chelation protocol with deferiprone (DFP) based on a, 6-month delayed-start paradigm, randomized placebo controlled clinical trial in 40 PD patients. CP-ferroxidase activity was determined in blood and CSF together with the D544E gene polymorphism (rs701753). Iron levels were determined by R2* MRI sequence and the motor handicap by the UPDRS motor score.

**Results:**

After 6 to 12 months of DFP treatment, greater reductions in SN iron levels and UPDRS motor scores were obtained in patients with higher serum and CSF levels of CP-ferroxidase activity. After 6 months of DFP treatment, the AT genotype group displayed greater reduction of iron level in the SN with greater CSF and serum levels of CP activity than the AA genotype group.

**Conclusion:**

Although most of the DFP-treated patients displayed clinical and radiological improvements, those with the lower CP activity appeared to respond better to iron chelation. Larger RCTs are now needed to establish whether pharmacological modulation of CP activity could be an innovative neuroprotective strategy in PD.

**Trial registration:**

FAIR-PARK study (ClinicalTrials.gov reference: NCT00943748; French national reference number: 2008−006842−25). This study was approved by the French Drug Agency (ANSM) and the local institutional review board (“Comité de Protection des Personnes of Lille”).

**Electronic supplementary material:**

The online version of this article (doi:10.1186/s12883-015-0331-3) contains supplementary material, which is available to authorized users.

## Background

The oxidative damage associated with extrahepatic siderosis is generally attributed to excessive generation of reactive oxygen species following a rise in labile cell iron [1,2]. The latter generally attains toxic levels as a result of imbalances between iron ingress, use, storage and egress. Cell iron egress and thereby cell iron accumulation appear to be modulated by ceruloplasmin (CP), a copper ferroxidase that facilitates the incorporation of plasma iron (II) into circulating apotransferrin. Ceruloplasmin’s key role is illustrated by an extreme example: that of aceruloplasminemia, which affects both systemic and brain iron metabolism. Low CP-ferroxidase activity has also been identified in the substantia nigra (SN) and the cerebrospinal fluid (CSF) of patients with Parkinson’s disease (PD) [3–5], and particularly in individuals bearing the AT genotype of the rs707753 CP gene variant (corresponding to a D544E protein change) [2]. A screening of ceruloplasmin gene sequences variations allowed to identify five new missense variations and an already known variation (rs707753), which was not previously identified in PD. Of these six missenses variations, D544E polymorphism was the most frequent and the only one to be significantly associated with PD and iron overload measured in SN by transcranial ultrasound [6]. Although a growing body of evidence suggests that PD is associated with oxidative damage via iron accumulation in the SN [7], CP’s putative involvement in iron accumulation and disease progression remains subject to debate. One way of addressing this issue involves comparing the ability of moderate iron chelation treatment to reducing both SN iron levels and United Parkinson’s Disease Rating Scale (UPDRS) scores in PD patients according to the level of ceruloplasmin activity. The underlying rationale is that PD patients with lower CP activity might retain more cell iron and thus respond more favorably to iron chelation therapy.

## Methods

To test this hypothesis, we used a moderate chelation protocol with deferiprone (DFP, administered at 30 mg/kg/day in two daily doses) that had previously served as the basis for a translational study (i.e. including a preclinical study with cells and mice models and the first double-blind, placebo-controlled, randomized clinical trial of an iron chelator in PD) [8,9].

### The FAIR-PARK study

(ClinicalTrials.gov reference: NCT00943748; French national reference number: 2008 − 006842 − 25) was based on a 6-month delayed-start paradigm in 40 PD patients on stable dopamine regimens. Treatment with DFP was initiated at 0 or 6 months and stopped at 18 months. In the initial phase, 40 early-stage PD patients were randomly assigned to receive either oral liquid DFP (15 mg/kg in the morning and evening) for 18 months (the ES group) or placebo for 6 months and then DFP for 12 months (the DS group) (Table 1).

**Table 1:** **Neurological characteristics of the patients on study entry (overall and by genotype, AT vs. AA)**

	**Delayed-start group**	**Early-start group**		
	**Total**	**Total**	**AT genotype**	**AA genotype**
Number of patients	19	21	5	14
Age at study entry (years)	60 [54–64]	61 [54–66]	62 [61–62]	61 [53–66]
Gender (F/M)	6/13	9/12	3/2	6/10
Time since diagnosis (years)	2 [1–3]	2 [1–3]	2 [1–3]	2 [1–3]
Hoehn and Yahr stage (range, 1–5)	2 [1.5-2]	2 [1.5-2]	2 [1.5-2]	2 [1.4-2]
Daily L-dopa dose equivalent (mg)	300 [162–510]	300 [150–532]	300 [150–532]	300 [150–532]
Patients on L-dopa (n)	9	10	3	7
Patients on dopaminergic agonists (n, mean daily L-dopa equivalent in mg)	18 (243)	18 (220)	4 (225)	13 (250)
Patients on L-dopa and agonists (n)	7	7	2	5
Patients on rasagiline (n)	3	3	1	2
Mini Mental Scale Examination score	29 [28–30]	28 [26–30]	28 [27–29]	28 [26–30]
Mattis Dementia Rating Scale score	139 [138–141]	139 [134–143]	139 [138–140]	139 [133–143]
Epworth Sleepiness Scale score	9 [7–13]	9 [5–13]	8 [6–10]	9 [5–13]
Montgomery Asberg Depression Rating Scale	3 [2–4]	4 [2–5]	3 [2–5]	4 [2–5]
Cognition and behavior: MDS-UPDRS part I score	6 [5–8]	6 [4.7-9]	5 [4–7]	6 [4.7-9]

There were no significant intergroup differences. The dopaminergic drug regimens did not change over the course of the study. Quantitative variables are quoted as the median [interquartile range]. MDS means Movement Disorders Society.

### Study approval

All clinical investigations were performed in accordance with the tenets of the Declaration of Helsinki. All patients provided their written, informed consent to participation. The aims and procedures of the main study and a compassionate 12-month extension was approved by the French Drug Agency (ANSM) and the local institutional review board (“Comité de Protection des Personnes of Lille”).

### Motor handicap

(i.e. disease progression) was assessed using the UPDRS motor score.

### Magnetic resonance imaging

Was performed with multiple-echo/spin-echo sequences in a 3-Tesla MRI system, in order to estimate the R2* proton relaxation rates (=1/T2*) and thus levels of agglomerated iron in a surface analysis of the SN (performed with the RelaxMaps tools in the Philips Research Imaging Development Environment®.

### The sample size

Was based on R2* measurements in the SN. We estimated that with an alpha risk of 5% and a power of 80%, 24 subjects per treatment group would be needed to detect a change in SN R2* after 6 months of DFP treatment (relative to placebo). When the correlation coefficient between consecutive measurements (at 6-month intervals) was set to 0.4 and the data were adjusted for baseline R2* levels, the required sample size fell to 20.

### Clinical biochemistry assays

Included ferritin, CP and CP-ferroxidase activity in blood and CSF. Levels of CP-ferroxidase activity in CSF and sera were determined according to the method published by Schosinsky et al. (1974) [10], as modified by Martinez-Subiela et al. (2007) [11] and Siotto et al. (2014) [12]. Briefly, the procedure is based on the CP-mediated oxidation of o-dianisidine, a chromogenic substrate forming a yellow-brown reaction product that absorbs at 500 nm. The CP activity is proportional to the rate of formation of reaction product. The working reagent solution was prepared immediately before use by mixing 1 volume of substrate solution (o-dianisidine dihydrochloride: 7.8 mM, Triton X-100: 1% v/v) with 4 volumes of sodium acetate buffer solution (100 mM, pH adjusted to 4.6). All reagents were from Sigma-Aldrich. Samples of CSF or serum (15 μL) were added to 300 μL of working reagent solution and the increase in absorbance at 500 nm over 100 s (first reading point: 200 s; second reading point: 300 s) at 37°C was read [11]. The following parameters were used to provide the results in international units per liter (IU/L), parameters we used were as follows: molar absorption coefficient of o-dianisidine oxidation product at 37°C (ε500 nm) = 4.83x10 [3] L/mol/cm, optical path length = 1 cm, dilution factor = 2 1, and change in time = 100 s. Reference levels of CP activity were defined using in-house standard CSF and serum samples, which were included as controls in all assays.

#### Genetic analysis

Genomic DNA was extracted from venous blood samples using standard procedures. The rs701753 CP gene variant (corresponding to a D544E protein change) was genotyped using a polymerase chain reaction (PCR)-restriction length polymorphism assay. The total reaction volume of 25 μl contained 50 ng genomic DNA in AmpliTaq PCR buffer with one unit of ampli Taq polymerase (Applied Biosystems, CA, USA), 1.50 mM MgCl_2_, 250 μM dNTPs, and 15 pmol of forward primer (5’-CCCCAGTTGGACTTACCTGT-3’) and reverse primer (5’-GATCCTGTGTGTCTAGCTAAGATG-3’). The cycling conditions were as follows: 5 min at 96°C, 35 cycles of 30 sec at 94°C, 1 min at 55°C and 1 min at 72°C, and then 5 min at 72°C. Amplification products were resolved in 5% acrylamide gels. BamH1 endonuclease was used to characterize the A and T alleles.

### Statistical analysis

In view of the sample size and the skewed, all quantitative variables were expressed as the median (interquartile range).

We used a covariance analysis (adjusted for baseline differences) to estimate outcomes after 6, 12 and 18 months of treatment. For non-normally distributed data, the robustness of the results was checked after log transformation. The threshold for statistical significance was set to p < 0.05. We used Wilcoxon’s signed-rank test to compare a pair of related measurements (e.g. repeated measurements on a single sample). All statistical analyses were performed with SAS software (version 9.3, SAS Institute Inc., Cary, NC).

## Results

**Figure 1:**
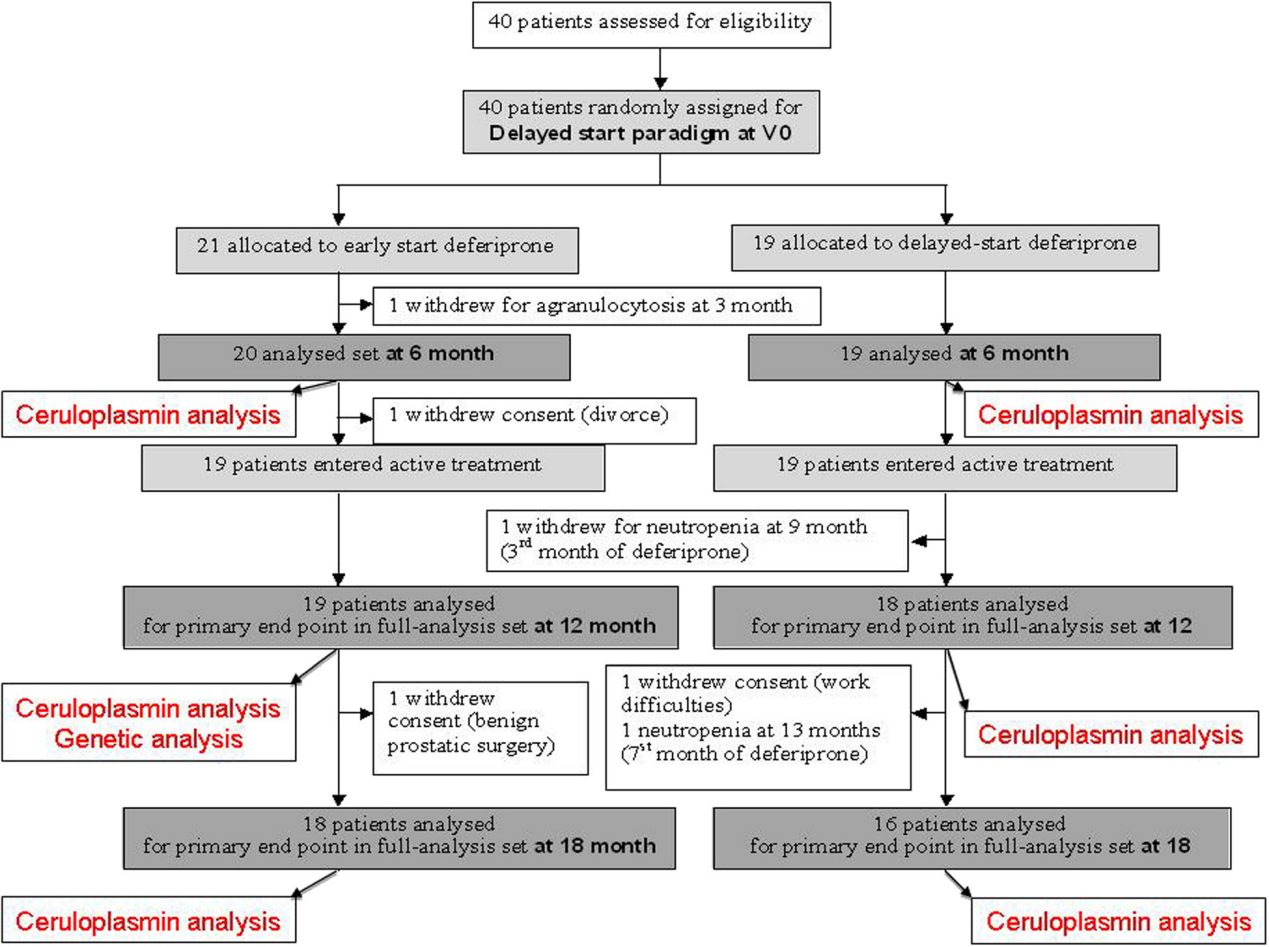
Flowchart of the patients who participated to the clinical trial and to the ceruloplasmin analysis.

**Figure 2:**
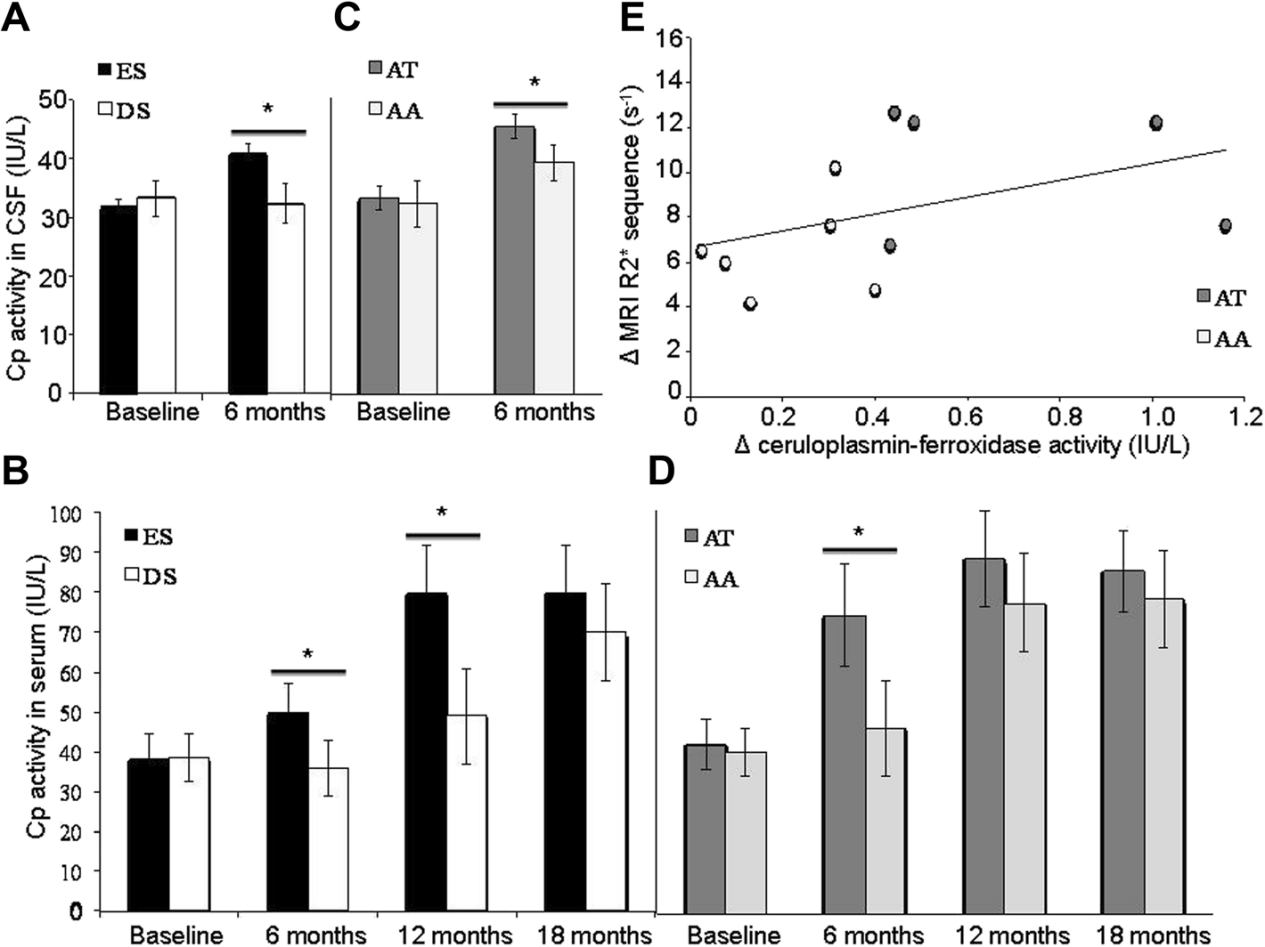
Ceruloplasmin activity and iron chelation treatment. **(A)** Effect of the iron chelator DFP on CSF levels of CP-ferroxidase activity. The patients in the ES group (n = 11 with two lumbar punctures: at baseline and at 6 months) displayed significantly higher CSF levels of CP activity than the patients in the DS group (F_(1,16)_=13; p=0.002 **(B)** Effect of the iron chelator DFP on serum levels of CP-ferroxidase activity. The patients in the ES group showed significantly higher serum levels of CP activity than the patients in the DS group at 6 months (F_(1,35)_=26; p=0.0001) and 12 months (F_(1,34)_=5.2; p=0.028) but not at 18 months. **(C)** Effect of the iron chelator DFP on CSF levels of CP-ferroxidase activity as a function of D544E genotype. The DFP-treated patients with an AT genotype (n = 5) displayed significantly higher CSF levels of CP activity than the DFP-treated patients with an AA genotype (n = 6) (F_(1,8)_=7; p=0.02). **(D)** Effect of the iron chelator DFP on serum levels of CP-ferroxidase activity as a function of D544E genotype. The DFP-treated patients with an AT genotype (AT; n = 5) displayed significantly higher levels of CP activity than DFP-treated patients with an AA genotype (AA; n = 15) at 6 months (F_(1,17)_=7; p=0.02) but not at 12 or 18 months. **(E)** Correlation between CSF levels of CP-ferroxidase activity and the R2* MRI value in the SN. The change in CSF levels of CP activity between the baseline and the visit at 12 months were significantly correlated with the change in the SN’s R2* value (r=0.784; p=0.001). White circles represent the DFP-treated patients with an AA genotype. Grey circles represent the DFP-treated patients with an AA genotype with an AT genotype; the latter displayed higher levels of CP activity and a greater reduction in R2* in the SN.

Deferiprone reduces the abnormally high levels of iron in the SN (with little effect on systemic iron levels defining a conservative mode of iron chelation [8]) and the UPDRS motor scores (i.e. indicators of disease progression) [9]. We compared results obtained in patients having started DFP treatment (30 mg/kg/day) early (the ES group; Figure 1) with those with a delayed start (the DS group; Figure 1). We observed that after 6 to 12 months of treatment, relatively greater reductions in SN iron levels and UPDRS motor scores were obtained in patients with higher serum and CSF levels of CP-ferroxidase activity (Figure 1, Figure 2 A&B). The increase in serum CP-ferroxidase activity was associated with significant higher serum CP levels after 12 months of DFP treatment in the ES group (from 0.28 g/L [0.26-0.32] to 0.30 g/L [0.26-0.35] as compared with the DS group (from 0.27 [0.24-0.29] to 0.27 [0.24-0.30]; p = 0.04).

We screened for the D544E gene polymorphism (rs701753: AT and AA genotypes) in the ES group; after 6 months DFP treatment, the AT group displayed greater CSF and serum levels of CP activity than the AA group (Figure 2 C & D). On the basis of median [interquartile range] R2* values in MRI (Figure 2 E), iron levels in the SN fell greater in the AT group (from 4.5 [4.2-4.9] at baseline to 3.9 [3.8-4.1] after 6 months (F_(1,17)_ = 7.9; p = 0.01) and 3.8 [3.6-4] after 12 months (F_(1,16)_ = 10.1; p = 0.06)) than in the AA group (from 4.6 [4.4-4.8] at baseline to 4.4 [4.2-4.4] after 6 months and 4.0 [3.8-4.3] after 12 months).

Although the AT group displayed a greater mean reduction in the motor UPDRS score than the AA group (decreases of 4 and 3 points, respectively), the difference was not statistically significant.

## Discussion

Our results suggest that iron chelation efficacy appeared to be related with the level of CP activity. PD patients with lower CP activity might retain more cell iron and thus respond more favorably to iron chelation therapy. Thus, CP may have a pivotal role in iron accumulation, which exacerbates the PD progression. Although most of the DFP-treated patients with PD displayed clinical and radiological improvements, those with the D544E CP polymorphism appeared to respond better to iron chelation. The greater mean reduction in the motor UPDRS score in the AT group as compared with the AA group did not reach the significant level. This might have been related to the small sample size and the many factors affecting the clinical response (notably DFP’s symptomatic effect through inhibition of catechol-O-methyl transferase) [9].

Larger RCTs are now needed to establish whether (i) the D544E CP polymorphism is a bona fide biomarker for responsiveness to iron chelation therapy in selected PD patients and (ii) pharmacological modulation of CP activity, by itself, could be an innovative neuroprotective strategy in PD.

## Conclusion

Lower ceruloplasmin activity in PD may be associated with iron overload in SN. Ceruloplasmin ferroxidase activity deficiency in PD could be partly improved by iron chelation by deferiprone in both groups (AA and AT) but mostly in the AT group, in which D544E polymorphism induced a lower ferroxidase activity. The measure of the R2* MRI sequence in SN was improved in both groups but the reduction was higher in the D544E (AT).
